# Body Mass Index Instability and Incident Mild Cognitive Impairment Among Black Older Adults

**DOI:** 10.1093/geroni/igaa057.1660

**Published:** 2020-12-16

**Authors:** Adrienne Aiken-Morgan, Ana Capuano, Zoe Arvanitakis, Lisa Barnes

**Affiliations:** 1 North Carolina A&T State University, Greensboro, North Carolina, United States; 2 Rush University Medical Center, Chicago, Illinois, United States

## Abstract

Despite general negative health effects, elevated body mass index (BMI) can be “protective” against poor health outcomes, including cognitive decline and mild cognitive impairment (MCI), in old age. However, few studies have examined the effects of BMI fluctuations (BMI instability) over time. The purpose of this study was to examine how BMI level at baseline and BMI instability is related to incident MCI in Black participants from the Minority Aging Research Study (MARS; N = 522, mean age = 73.5, mean education = 15.0; 76.5% women). Participants without cognitive impairment at baseline underwent annual clinical evaluations, including measurement of BMI and 19 neuropsychological tests for up to 15 years of follow-up to document MCI. 192 of 522 persons developed MCI. In Cox models adjusted for age, sex, and education, 1) higher baseline BMI, across the range of all values (mean=30.5; SD=6.5), was related to a decreased risk of MCI (Hazard Ratio = 0.97; 95% CI = 0.94-1.00); and 2) BMI instability (with a maximal range of 0-15.7; mean=3.2; SD=2.5) was related to an increased risk of MCI (Hazard Ratio = 1.09; 95% CI = 1.03-1.15). The present findings suggest that while late-life higher BMI level may protect against MCI, BMI instability over the years is detrimental to cognition in Black persons without dementia. Future research should investigate underlying mechanisms.

